# Economic Considerations for Moving beyond the Kato-Katz Technique for Diagnosing Intestinal Parasites As We Move Towards Elimination

**DOI:** 10.1016/j.pt.2017.01.007

**Published:** 2017-06

**Authors:** Hugo C. Turner, Alison A. Bettis, Julia C. Dunn, Jane M. Whitton, T. Déirdre Hollingsworth, Fiona M. Fleming, Roy M. Anderson

**Affiliations:** 1 London Centre for Neglected Tropical Disease Research, London, UK; 2 Department of Infectious Disease Epidemiology, School of Public Health, Faculty of Medicine, St Marys Campus, Imperial College London,Norfolk Place, London W2 1PG, UK; 3 Oxford University Clinical Research Unit, Wellcome Trust Major Overseas Programme, Ho Chi Minh City, Vietnam; 4 Centre for Tropical Medicine and Global Health, Nuffield Department of Medicine, University of Oxford, Oxford, UK; 5 Schistosomiasis Control Initiative, Department of Infectious Disease Epidemiology, School of Public Health, Faculty of Medicine (St Mary’s Campus), Imperial College London, Norfolk Place, London W2 1PG, UK; 6 Mathematics Institute, University of Warwick, Coventry CV4 7AL, UK; 7 School of Life Sciences, University of Warwick, Coventry CV4 7AL, UK

## Abstract

While the need for more sensitive diagnostics for intestinal helminths is well known, the cost of developing and implementing new tests is considered relatively high compared to the Kato-Katz technique. Here, we review the reported costs of performing the Kato-Katz technique. We also outline several economic arguments we believe highlight the need for further investment in alternative diagnostics, and considerations that should be made when comparing their costs. In our opinion, we highlight that, without new diagnostic methods, it will be difficult for policy makers to make the most cost-effective decisions and that the potentially higher unit costs of new methods can be outweighed by the long-term programmatic benefits they have (such as the ability to detect the interruption of transmission).

## Trends

The Kato-Katz technique is a widespread tool in intestinal helminth epidemiological surveys. It is used in monitoring and evaluation programmes to investigate the prevalence and geographical distribution of soil-transmitted helminth and *Schistosoma mansoni* infections – determining the WHO treatment strategy to be used.

While the need for more sensitive diagnostic methods for these intestinal parasites has been identified, the cost of developing and employing new and potentially more rapid tests is considered relatively expensive, particularly compared to the Kato-Katz technique (which is often referred to as cheap). Consequently, this area has suffered from a lack of investment.

The actual cost of using the Kato-Katz technique is higher and more variable than is often being assumed, and it is difficult to implement at a large scale.

## The Kato-Katz Technique

The World Health Organization (WHO) recommends using the **Kato-Katz technique** [1] (see Glossary) for diagnosing and quantifying **soil-transmitted helminth** (STH) and ***Schistosoma mansoni*** infections – as it provides a standardized reading (eggs per gram of faeces (epg)) and the technique can be taught to laboratory microscopists relatively easily. It is performed using a small spatula and a slide template that allows a standardized amount of faeces to be examined under a microscope and the eggs to be counted. Though the technique can be done in the field without sophisticated laboratory techniques or equipment, it is labour intensive and requires expertise in microscopy.

The Kato-Katz technique is a widespread tool in intestinal helminth epidemiological surveys. It is used in monitoring and evaluation programmes to determine the geographical distribution of STH and *S. mansoni* infections which concomitantly defines the endemicity of an area before control (determining the treatment strategy to be used) 2, 3. Furthermore, the Kato-Katz technique is used in post-treatment surveys to assess the impact of control by measuring changes in the prevalence and intensity of infection. However, the technique has a poor sensitivity for detecting light intensity infections, and there is substantial variation in the readings (resulting from day-to-day fluctuations of egg excretion, uneven distribution of eggs within a single stool sample, and variation in the ability of the technicians) 4, 5, 6, 7, 8, 9, 10, 11. Consequently, it is recommended that the test be performed in duplicate, with two slides prepared and read per sample [12]. Additional challenges involved with STH include the need to collect and process fresh stool samples within a limited timeframe (as hookworm eggs start to degrade rapidly within 30min of sample preparation 3, 13), which adds logistical constraints to Kato-Katz-based surveys.

In this paper, we review the published costs of performing the Kato-Katz technique, and examine the variation in the reported values. We also outline several economic arguments that, in our opinion, support further investment in alternative diagnostic tools, particularly as the overarching policy for these diseases shifts from morbidity control to transmission elimination.

## Costs of Performing the Kato-Katz Technique

A single Kato-Katz kit, which includes the template and plastic spatula, only costs between 0.1 and 0.3 US$ [14]. These kits are typically washed and then re-used over multiple collection sites and surveys. Consequently, other than the initial investment in a microscope, the Kato-Katz technique is a cheap test in terms of the required materials and equipment. However, because the technique requires stool samples to be collected and processed, it is associated with a significant personnel cost. This is due to the time required to collect and prepare stool samples as well as the number of staff needed to conduct the survey due to the logistical demands of the technique. As a result, personnel-related costs are often the largest contribution to the total cost of performing the Kato-Katz technique (for example, Speich *et al.* [14] found that personnel constituted 74% of the cost of performing a duplicate Kato-Katz test in a costing study).

**Figure 1:**
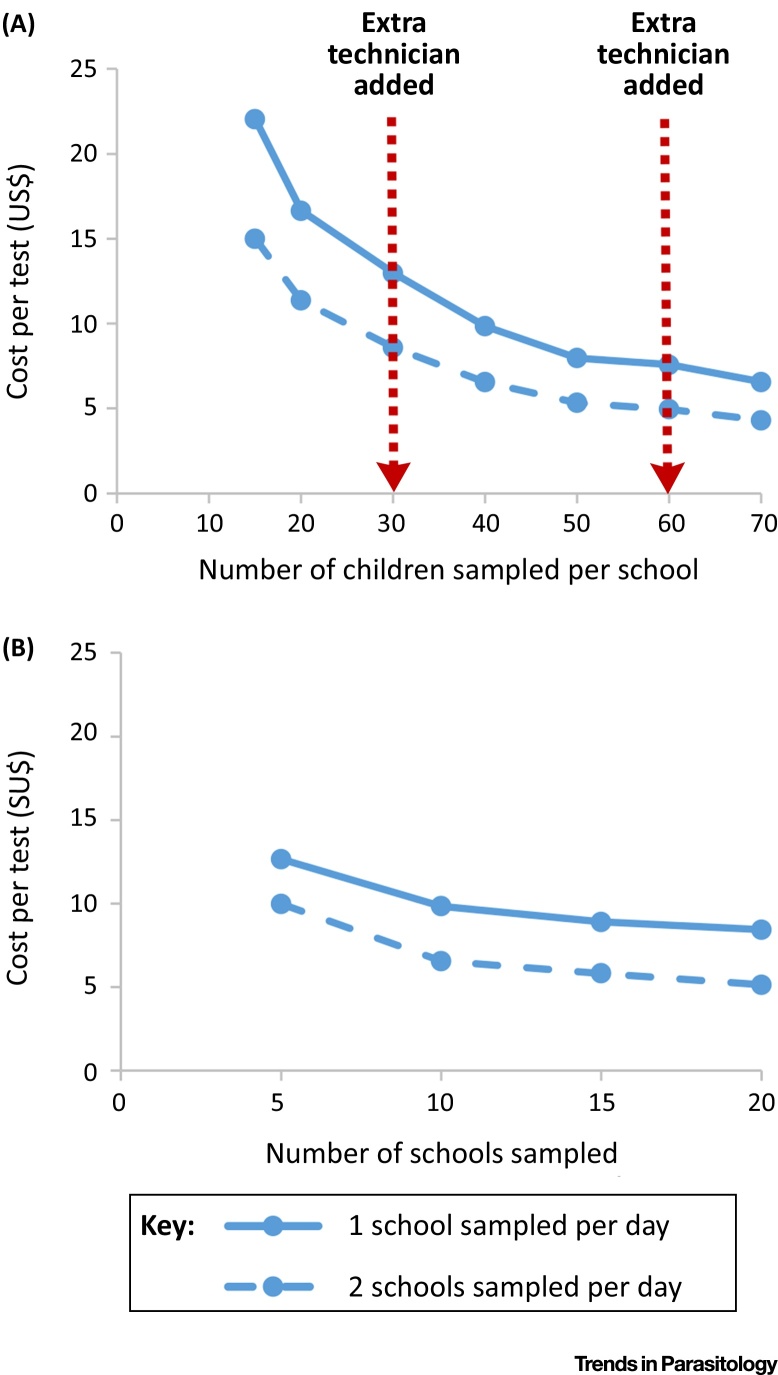
Economies of Scale in School-Based Kato-Katz-Based Surveys. These figures are based on the itemized costs for performing a Kato-Katz-based survey in Ethiopia presented by Sturrock *et al.*^16^, ^17^. They relate to the use of a mobile mapping team (who collect and process the samples at each survey site). Panel (A) assumes that 10 schools were sampled in the survey. Panel (B) assumes that 40 children were sampled per school. Our analysis assumes that 2 days are spent travelling to/from the field site and account for no samples being able to be collected on weekends. The costs are expressed in 2009 US$ prices.
(iii) Variation in personnel costs. There is variation in the reported country-specific daily per diems for laboratory technicians {e.g., 14.29 US$ in Kenya versus 37.23 US$ in Ethiopia (2009 prices) [17]}. The personnel costs are often the main driver in the cost of performing the Kato-Katz technique, and can cause substantial variation in the test’s costs between different settings. The majority of the identified studies reported only the **financial costs** of personnel (i.e., the staff’s per diems). If the **opportunity cost** (economic value) of the staff’s time is included in the analysis [15], the costs associated with personnel would be even larger (**economic costs**).
(iv) Adjustment of microscope costs. If the microscope is also used for other activities, only a percentage of the total cost would be applied to the Kato-Katz-based survey, thus, reducing the cost per test. Furthermore, the cost of a microscope is often annualized (meaning the cost is spread over its useful lifetime). If this is not performed, the cost per test would be higher.
(v) Number of samples taken. There are **economies of scale** associated with Kato-Katz-based surveys (as the sample size is increased, the cost per test typically decreases) (Figure 1). Consequently, the sample size should be considered when comparing the cost per test reported from different studies/settings [17].

The reported cost of performing a duplicate Kato-Katz test varies between 2.67 and 12.48 US$ [adjusted to 2014 prices (https://www.imf.org/external/pubs/ft/weo/2016/02/weodata/index.aspx)] in the published literature 14, 15, 16, 17, 18. This variation is can be attributed to several key factors:

(i) Method of collection. In some settings sample pots are left overnight, and then taken to a local laboratory facility for processing the next day [14], whereas others use a mobile team of technicians which collect and process the samples at each survey site 16, 17.
(ii) Number of sites sampled per day (Figure 1) and the distance between them.

A further source of potential variation in the reported costs is the level of community sensitization that has been performed. It should be noted that though performing community sensitization is an extra cost, it can make it easier and quicker to obtain the samples. This will be particularly important for studies/settings that are also sampling from adults (as opposed to only children in schools) and the amount needed (and its cost) will depend on the local culture.

### Economies of Scale in Kato-Katz-Based Surveys

Within a Kato-Katz-based survey, increasing the number of study participants sampled per site results in economies of scale (Figure 1), as many of the costs are **fixed/step-fixed** and incurred regardless of the number of samples processed at each site. Consequently, the cost per test is not constant. We illustrate this based on cost data collected in Ethiopia 16, 17. However, the reduction in the cost per test approaches a limit (Figure 1). This is because, as the sample size increases, there are thresholds above which more technicians would need to be employed in order to process all samples collected that day 16, 17. Consequently, there is a limit to what degree economies of scale can reduce the cost of Kato-Katz-based surveys (Figure 1). These sample size thresholds may vary depending on the expertise of the technicians, and the distance between the collection sites.

In contrast to increasing the number sampled per sample site, increasing the number of sites sampled within a Kato-Katz-based survey is not associated with the same degree of economies of scale (Figure 1). This is because sampling more sites takes longer, and therefore increases the total personnel and transportation costs of the survey. In other words, many of the costs do not change (i.e., they are fixed) with regard to the number sampled at each site (generating economies of scale when increasing the number sampled within each school), but do change in relation to the number of sites sampled (stepped-fixed costs). The degree of economies of scale will vary in different settings (Figure 1). For example, there may be greater economies of scale in settings where the samples are brought to a local laboratory facility rather than processed at each school or in settings that have lower per diems for the technicians.

It should be noted that these costs could be reduced by integrating the Kato-Katz technique within another survey (such as transmission assessment surveys (TAS) for lymphatic filariasis 19, 20), resulting in so-called **economies of scope**.

This analysis highlights that the unit cost of performing the Kato-Katz technique will vary depending on sample size and number of sample sites. Therefore, the cost per test should be not be considered to be constant for different study designs and study sites.

## Economic Arguments for New Diagnostic Methods

The need for more sensitive diagnostic methods for the STH and schistosomes (SCH) has been frequently identified in recent years 21, 22, 23, 24, 25, 26, and there have been recent advances in the development of potential alternatives 9, 23, 27, 28, 29, 30, 31. However, these methods are often considered expensive, and they are rarely used within neglected tropical diseases (NTD) control programmes at present. Investment is lacking to make many of these new tests more suitable for mass implementation in the field or for the development of other novel methods [23] (http://www.policycures.org/downloads/g-finder_2010.pdf).

In the following section, we outline several economic arguments we believe highlight the need for further investment in alternative diagnostic methods to the Kato-Katz technique, particularly as the overarching goal for these diseases shifts from morbidity control to transmission interruption and elimination. It is important to highlight that the requirements/ideal features of a test are dependent on the stage of the programme (for example, the features of a test ideal for conducting pre-control mapping are not the same as those ideal for certifying elimination/post-mass drug administration (MDA) surveillance 21, 22, 26). We therefore focus our economic arguments on two key areas (i) more rapid and convenient tests, and (ii) more sensitive tests (though these should not be treated as mutually exclusive as it is possible that a test could have both of these qualities).

### Economic Arguments for More Rapid and Convenient Tests

#### Reduced Implementation Costs for Conducting Epidemiological Surveys

Though it is likely that a new test or method will be more expensive in terms of the required materials, they can, in some cases, be faster to process and prepare – particularly if they do not require a stool sample. For example, the unit cost of a urine-based point-of-contact circulating cathodic antigen (CCA) test alone is now around 1.46 to 1.76 US$ [18] (which is higher than the unit cost of a Kato-Katz kit), but the test is much faster and less labour intensive to implement [32]. Consequently, a survey using the CCA test instead of the Kato-Katz technique could potentially be performed faster and with fewer staff, reducing the personnel cost required to implement the survey. Furthermore, if the staff do not require training in microscopy to perform the diagnostic test, scaling-up implementation would be more programmatically feasible and cheaper (it is important to note that the number of trained parasitologists can be limited in some endemic areas). This is illustrated by the study by Worrell *et al.* [18] who found that the labour costs associated with using the point-of-contact CCA test were less than that of the Kato-Katz test. Ideally, a new diagnostic method could be performed by community health workers and teachers, and therefore integrated within the distribution of treatment, at a much lower cost than if trained laboratory personnel were required (though the opportunity cost of this should be evaluated). It should be noted that a recent study found that pooling of stool samples may be a way of increasing the speed of the Kato-Katz technique [33]. However, how this method affects the cost of performing the Kato-Katz technique and its sensitivity at low infection levels requires further investigation.

Past research has shown that it is more effective and accurate to increase the number of schools sampled within a pre-control mapping survey compared to sampling more children per school [16]. Point-of-contact rapid diagnostics would make this more feasible and achievable at a potentially lower cost. Furthermore, if the method had a greater sensitivity than the standard Kato-Katz technique, a smaller sample size would be required to have the equivalent statistical power – again bringing down the costs. When considering potential samples sizes, it is important to recognise that, as we progress towards elimination, the decrease in infection prevalence will necessitate a larger sample size than previously used to retain statistical power. Furthermore, if a new test had a higher sensitivity than the Kato-Katz technique, it would result in an increase in the reported prevalence of infection. Calibration will be essential to allow measurements from new methods to be equated to past Kato-Katz (epg) measures [31]. This is particularly important when interpreting WHO’s treatment guidelines (which are based on prevalence estimates obtained using the Kato-Katz technique).

#### Informing Treatment Strategies and Expanding Sampling beyond Schools

Due to financial and programmatic constraints, the majority of Kato-Katz-based surveys for STH and *S. mansoni* currently focus on sampling children in schools. However, this provides a misleading picture regarding the impact of treatment on the overall rate of transmission and infection levels in the community as a whole [34]. Mathematical modelling studies have indicated that, in many settings, we need to consider expanding interventions beyond the school to include the community as a whole (or for *Trichuris trichiura,* to increase the treatment frequency and/or use combination therapy) 34, 35, 36, 37, 38, 39, 40. Changes in control strategies will be particularly important as goals gradually shift from morbidity control to eliminating transmission 34, 40, 41. As it will very likely not be possible to implement these more intensive strategies in all STH/SCH endemic areas, knowing the age and species-specific burden in the overall community will be crucial to informing MDA policy and prioritizing which areas need to move to community-wide treatment. An obvious example is the control of hookworm, where most infection is harboured by adults [38]. Without accurate information regarding the infection prevalence in the community (as well as the schools), it will be very difficult for NTD programmes to make the most cost-effective policy decisions. This could lead to an inefficient use of resources and make eliminating transmission impossible (which is potentially very costly in the long term [42]). In this context, Kato-Katz-based surveys would be very expensive (due to the time it would take to collect stool samples from the community, and the level of community sensitization needed to increase participation by adults), so more rapid and accurate diagnostics would be preferable. Any new STH diagnostic method would ideally be able to identify the three major STH species separately (allowing the optimum intervention to be targeted to the local setting 34, 35, 36, 37, 38).

#### Potential Need for a ‘Test and Treat’ Strategy in Areas Close to Achieving Elimination

As specific settings move closer to elimination, it may be necessary to consider using a selective treatment strategy in some areas (where only individuals that are infected (‘test and treat’) [22] or only those predisposed to reinfection are treated). This would reduce the volume of drug treatments being used in areas where the prevalence is very low, allowing them to be reallocated for more intensive treatment in areas where progress is behind target (which will be important as we approach the maximum capacity of the drug donation programmes). However, a more rapid and sensitive diagnostic is needed for this approach to be an economically feasible possibility.

#### Integration of Sample Collection with Other NTD Surveillance Programmes

Recent guidelines have recommended the coordination of various NTD surveillance surveys (such as STH and lymphatic filariasis [43]). However, the need for stool collection – as opposed to blood or urine – makes this more challenging [44] and costly. A more rapid and convenient test would make this integration more feasible.

### Economic Arguments for More Sensitive Tests

#### Confirmation of Elimination and Detecting Resurgence

Accurate diagnostics are needed to confirm when and where transmission has been interrupted and to detect infection resurgence quickly. However, due to the limited sensitivity of the Kato-Katz technique at low infection levels 4, 5, 6, 45, it is unlikely to be suitable for this, and a more sensitive test will be required 9, 22, 28, 45, 46.

When considering the need for, and value of, these higher sensitivity tests, it is important to note that if we are unable to confirm when and where transmission has been interrupted, it could significantly increase control programme costs due to overtreatment. This would mean that the potential long-term cost-savings of using more intensive elimination strategies (such as community-wide treatment) could be significantly diminished 34, 38. Consequently, even if the unit cost of the new method is higher than that of the current Kato-Katz technique, it may still be cost-saving in the long term if it allows treatment to be stopped at the right time and resurgence to be detected – as it could reduce unnecessary overtreatment and allow the control of infection resurgence before its spread to other areas (preventing the costs of having to restart control programmes).

#### Detecting the Emergence of Drug Resistance

More sensitive diagnostic methods would also be useful for detecting any development of anthelmintic resistance [47]. Drug resistance has already been observed by the veterinary community when using the same anthelmintics in the treatment of animals, which further highlights the urgency for developing such tests [48]. Early detection of the development of drug resistance in human infections will be vital for ensuring that appropriate management strategies are implemented to reduce its spread – which could have important economic consequences for the control programmes.

## Comparing the Costs and Cost-Effectiveness of New Methods

When investigating the costs and cost-effectiveness of alternative tests, it will be important to consider that their costs will vary across different programmatic settings (as well as when they are performed in different contexts, e.g., a scientific study versus an NTD surveillance programme). In particular, any cost-savings resulting from the use of more rapid tests will be very sensitive to the increase in number of schools/survey sites that can be sampled per day (highlighted by the different curves in Figure 1 and [18]), and therefore dependent on the population density of the survey area. Furthermore, the relative costs of different tests will also vary depending on the sample size (as the amount of economies of scale relating to different tests may not be the same (Figure 1). In addition, the cost of purchasing novel tests is likely to decrease over time as demand for them increases [49] (as has been observed for the CCA test [18]). These factors have significant implications regarding the generalisability of cost data and for the conclusions drawn regarding the relative costs of different diagnostic methods. This highlights the need for us not to overgeneralise the cost data of new tests and the importance of accurately capturing their implementation costs – such that we can understand what is driving the relative costs of doing different tests. When comparing the costs of different tests from different studies, the use of costing models will be advantageous and allow for a more accurate comparison of the cost estimates (and their potential uncertainty or generalisability).

It is important to highlight that new tests will not necessarily always be more cost-effective than using the Kato-Katz technique, and this will depend on the local context and stage of the programme 21, 22, 26.

New tests that still require a stool specimen will likely have similar implementation costs to the Kato-Katz technique. However, if they have an increased accuracy they could still have important implications and programmatic benefits (particular regarding confirmation of elimination, and detecting resurgence) that may outweigh the extra costs in the long term. The complexity regarding evaluating/quantifying these long-term benefits has interesting parallels to the field of antimicrobial resistance and the evaluation of tests/interventions aimed at reducing the spread of resistance [50] (i.e., justifying an initial investment in more expensive strategies that prevent future losses occurring).

When considering the cost and cost-effectiveness of new methods it is important to note that in coendemic areas, STH and *S. mansoni* infections are often sampled together using the Kato-Katz technique. Therefore, in these areas, in order to gain an increase in programmatic feasibility and potential cost savings, improved tests would be needed for both diseases. Potential quality assurance methods (and their costs) should be considered when evaluating new tests.

## Concluding Remarks

Though the development and implementation of novel diagnostic methods are considered expensive, as our global goals move towards elimination we believe that there are strong economic arguments for further investment in alternative diagnostic methods for intestinal parasites – particularly when considering that the reported costs of using the Kato-Katz technique are higher and more variable than is often assumed, and the fact that it is often difficult to implement at a large scale.

When investigating and comparing the costs of the new tests, it will be vital to consider that they are likely to vary across different programmatic settings and that the cost per test will not be constant. This highlights the importance that we accurately capture the implementation costs of performing new tests (particularly relating to personnel), and investigate what the key drivers in their total costs are. Doing this will allow us to understand how generalizable the costs/cost-effectiveness of new tests are to other settings, and will ensure that we have a scientific understanding of the relative costs of performing different tests (see Outstanding Questions).

In our opinion, this work also asserts that when comparing diagnostic methods, it is important to consider that potentially higher costs of new tests can be outweighed by the long-term programmatic benefits they may have. For example, the Kato-Katz technique has a poor sensitivity at low infection intensities and is therefore unlikely to be suitable for confirming transmission interruption. Without new diagnostic methods, it will be very difficult for STH and SCH control programmes to make the most cost-effective policy decisions, particularly as our goals shift towards elimination. The issues outlined regarding the economics of comparing diagnostic tests are of course highly relevant to the diagnostics tools for other diseases.

Outstanding Questions What are the costs of new tests, and how variable are they across different programmatic settings?

What is a cost-effective way to expand data collection from schools to the whole community?

How do results from more sensitive diagnostic test change the interpretation of the WHO treatment guidelines (which are based on results from the Kato-Katz technique)?

What test and survey design can be used to confirm the interruption of transmission and for detecting infection resurgence?

What is the most cost-effective method to monitor for the emergence of anthelmintic resistance?

## **Authors**’ Contributions

HCT drafted the first version of the manuscript. AAB, JCD, JMW, TDH, FMF, and RMA contributed to the design of the study and writing of the paper. All authors read and approved the final version of the manuscript.

## Glossary

**Economic costs**: economic costs represent the full value of all resources used (including for donated items for which no financial transaction has taken place). These are important when considering issues related to the sustainability and replicability of interventions.
**Economies of scale**: the reduction in the average cost per unit resulting from increased production/output: in this case the reduction in the cost per test as a result of increasing the number sampled.
**Economies of scope**: the reduction in the average cost per unit resulting from producing two or more products at once: in this case the reduction in the cost per test, when administering more than one test within the same survey (i.e., integrated control programmes).
**Financial costs**: these represent the accounting cost (i.e., actual amount paid) for a good or service.
**Fixed costs**: costs which are not dependent on the quantity of output (in this case, costs that are incurred and do not change regardless of the total number sampled).
**Kato-Katz technique**: the Kato-Katz technique is used for qualitative and semiquantitative diagnosis of intestinal helminthic infections. It is performed using a small spatula and slide template that allows a standardized amount of faeces to be examined under a microscope and the eggs to be counted.
**Opportunity cost**: the value of the benefit forgone of not being able to use a resource for its next best alternative use.
***Schistosoma mansoni***: Schistosomiasis (SCH) is caused by digenetic blood trematodes. *S. mansoni* is the most widespread of the human-infecting schistosomes.
**Soil-transmitted helminths (STH)**: soil-transmitted helminth infections are among the most common infections worldwide and affect the poorest and most deprived communities. The main species that infect humans are the roundworm (*Ascaris lumbricoides*), the whipworm (*Trichuris trichiura*) and the hookworms (*Necator americanus* and *Ancylostoma duodenale*).
**Stepped-fixed costs**: costs that are fixed for a particular level of activity/production, but increase incrementally once a threshold is crossed. For example, these costs can be fixed per school sampled, but are variable in terms of the number of schools sampled.
